# New Books

**Published:** 2008-11

**Biodiesel: Growing a New Energy Economy, 2nd ed.**

Greg Pahl

White River Junction, VT:Chelsea Green Publishing, 2008. 296 pp. ISBN: 978-1-933-39296-7, $19.95

**Biofuels, Solar and Wind as Renewable Energy Systems: Benefits and Risks**

David Pimentel, ed.

New York:Springer, 2008. 506 pp. ISBN: 978-1-4020-8653-3, $89.95

**Carbon and Nitrogen in the Terrestrial Environment**

R., Nieder, D.K. Benbi

New York:Springer, 2008. 432 pp. ISBN: 978-1-4020-8432-4, $159

**Carbon Nanotubes: Angels or Demons?**

Silvana Fiorito

Hackensack, NJ:World Scientific Publishing Co., 2008. 164 pp. ISBN: 978-981-4241-01-4, $109

**Global Warming: A Very Short Introduction, 2nd ed.**

Mark Maslin

New York:Oxford University Press, 2008. 176 pp. ISBN: 978-0-19-954824-8, $11.95

**Handbook of Toxicology of Chemical Warfare Agents**

Ramesh Gupta, ed.

St. Louis, MO:Elsevier, 2009. 1,300 pp. ISBN: 978-0-12-374484-5, $225

**Health Environment: Managing the Linkages for Sustainable Development**

World Health Organization/United Nations Environment Programme

Geneva:WHO Press, 2008. 86 pp. ISBN: 978-924-156372-7, $20

**Impacts on U.S. Energy Expenditures and Greenhouse-Gas Emissions of Increasing Renewable-Energy Use**

Michael Toman, James Griffin, Robert J. Lempert

Santa Monica, CA:Rand Corporation, 2008. 118 pp. ISBN: 978-0-8330-4497-6, $34.50

**Lake Effect: Two Sisters and a Town’s Toxic Legacy**

Nancy Nichols

Washington, DC:Island Press, 2008. 192 pp. ISBN: 978-1-59726-084-8, $24.95

**Large-Scale Ecosystem Restoration: Five Case Studies from the United States**

Mary Doyle, Cynthia Drew

Washington, DC:Island Press, 2008. 344 pp. ISBN: 978-1-59726-026-8, $35

**Natural Disaster Analysis After Hurricane Katrina: Risk Assessment, Economic Impacts and Social Implications**

Harry W. Richardson, Peter Gordon, James E. Moore II, eds.

Northampton, MA:Edward Elgar Publishing, Inc., 2008. 320 pp. ISBN: 978-1-84720-357-1, $160

**Poisoned for Pennies: The Economics of Toxics and Precaution**

Frank Ackerman

Washington, DC:Island Press, 2008. 352 pp. ISBN: 978-1-59726-401-3, $25

**Practising Science Communication in the Information Age**

Richard Holliman, Jeff Thomas, Sam Smidt, Eileen Scanlon, Elizabeth Whitelegg, eds.

New York:Oxford University Press, 2008. 264 pp. ISBN: 978-0-19-955267-2, $40

**Progress on Drinking-water and Sanitation**

World Health Organization

Geneva:WHO Press, 2008. 54 pp. ISBN: 978-924-156367-3, $15

**Protocells: Bridging Nonliving and Living Matter**

S. Rasmussen, M. Bedau, L. Chen, D. Deamer, D. Krakauer, N. Packard, P. Stadler, eds.

Cambridge, MA:MIT Press, 2008. 776 pp. ISBN: 978-0-262-18268-3, $75

**Science Magazine’s State of the Planet 2008–2009**

Editors of Science, Donald Kennedy

Washington, DC:Island Press, 2008. 216 pp. ISBN: 978-1-59726-405-1, $40

**Surviving 1,000 Centuries: Can We Do It?**

Roger-Maurice Bonnet, Lodewyk Woltjer

New York:Springer, 2008. 442 pp. ISBN: 978-0-387-74633-3, $39.95

**Sustainability by Design: A Subversive Strategy for Transforming Our Consumer Culture**

John R. Ehrenfeld

New Haven, CT:Yale University Press, 2008. 272 pp. ISBN: 978-0-300-13749-1, $28

**Tactical Biopolitics: Art, Activism, and Technoscience**

Beatriz da Costa, Kavita Philip, eds.

Cambridge, MA:MIT Press, 2008. 504 pp. ISBN: 978-0-262-04249-9, $40

**The Bridge at the Edge of the World: Capitalism, the Environment, and Crossing from Crisis to Sustainability**

James Gustave Speth

New Haven, CT:Yale University Press, 2008. 320 pp. ISBN: 978-0-300-13611-1, $28

**The Design of Climate Policy**

Roger Guesnerie, Henry Tulkens, eds.

Cambridge, MA:MIT Press, 2009. 408 pp. ISBN: 978-0-262-07302-8, $38

